# Testing the Feasibility of a Digital Point of Care Solution for the Trusted Near Real-Time Bidirectional Exchange of Novel and Informative Adverse Event Information

**DOI:** 10.1007/s43441-024-00711-9

**Published:** 2024-11-17

**Authors:** Greg Powell, Vijay Kara, Daniel Naranjo, Mangesh Kulkarni, Kerri Best-Sule, Trinka Coster, Machaon Bonafede, Shruti Gangadhar, Lee Kallenbach, Andrew Bate

**Affiliations:** 1 GSK, 5 Moore Dr, Durham, NC USA; 2 GSK, London, UK; 3 Pharmacocybernetics, LLC., Potomac, MD USA; 4 Veradigm Inc, Chicago, IL USA

**Keywords:** Adverse drug reactions, Adverse event reporting, Electronic health records, Real-world data, Digital health

## Abstract

A digital point-of-care solution was implemented to test the feasibility of near-real-time bi-directional communication between pharmacovigilance experts (PVEs) and healthcare professionals (HCPs) for exchanging unique and informative adverse event (AE) information. The solution was implemented in a commercially available electronic health record (EHR) system/platform, no direct contact between PVEs and the HCPs was possible. The Clinical Affairs team of the EHR vendor was used as an intermediary to ensure appropriate information was exchanged while protecting HCP and patient privacy. The study yielded 9 drug-event pairs of interest (AEI), 2 of which were confirmed as AEs by the HCP. On average it took 20.6 h to receive initial AEI information and 58.8 h to receive follow-up information, which represents a 96% reduction in time compared to current methods. Both interactions provided unique data that would not have been collected otherwise leading to the PVE being able to appropriately determine a potential causal association. This study successfully demonstrated the feasibility of using a compliant, bi-directional, digitally enabled clinical communication channel at the point of care to complement existing pharmacovigilance activities.

**Supplementary Information:**

The online version contains supplementary material available at 10.1007/s43441-024-00711-9.

## Introduction

Digital Medicine has been defined as using digital tools to upgrade the practice of medicine to one that is high-definition and far more individualized [1]. It is well established that digital tools can not only help lighten the burden on healthcare, but also improve patient outcomes by documenting and facilitating the exchange of important medical information (e.g., electronic medical records [2]. For example, a recently completed study showed that artificial intelligence based tools for reading magnetic resonance imaging (MRI) results for multiple sclerosis patients demonstrated superior case-level sensitivities as compared to standard radiology reports (93.3% versus 58.3%), which could lead to the detection of clinically silent new MRI lesions that would impact the treatment strategy [3].

Naturally, part of optimizing patient outcomes is understanding the benefits and risks of various treatment options. Once a drug has been launched, the cornerstone of post-marketing safety surveillance entails healthcare professionals reporting suspected AEs to regulatory agencies and pharmaceutical manufacturers. These reports are critical to protecting patients as demonstrated by the fact that between 1990 and 2010 they accounted for 65% of all drugs withdrawn from the market for safety reasons [4]. However, these reports have well-established limitations; under reporting (estimated to be 94%) [5–7], significant quality issues (less than 50% of the critical data elements are provided) [8, 9], lack of follow-up (13% will receive follow-up information) [10], and significant delays in gathering important information (average of 106 days) [10–12]. The current AE reporting process is still largely manual and increases the HCP burden. With the maturation of the digital environment used to facilitate the delivery of healthcare, such as cloud-based electronic health record (EHR) platforms, opportunities now exist to facilitate the systematic identification of potential AE and the rapid collection of the data to support causality assessment.

EHR data contains longitudinal information from patient visits, including structured (diagnosis, medication orders, procedures), semi-structured (lab results), and unstructured data (notes, reports, discharge summaries), data which can be critical for safety surveillance. Over the past several years, a number of studies have highlighted the potential value of digitizing the AE process (e.g., using electronic medical records, mobile applications, etc.). A study by Linder et al. (Project Aster) demonstrated that HCPs are more likely to report AEs via the use of electronic medical records: 21 of 23 respondents (91%) who submitted an AE report via the electronic medical record had not submitted any AE reports to the Food and Drug Administration (FDA) in the prior 12 months, and the time to complete the report was far faster than the current process (10 days on average) [11]. A study by Hoppe et al. found that leveraging mobile applications was helpful for clinicians to ascertain potential AEs in the emergency department setting and during inpatient follow-up for aseptic meningitis, encephalitis, myelitis, and acute disseminated encephalomyelitis [13].

Although previous studies provided an important step in demonstrating the potential value of digitizing elements of the AE process, there were several key limitations. While the systematic collection of data allowed for large quantities of highly complex data to be shared, the overall quality was low [11]. Brajovic et al. found that other aspects of reports, such as an informative description of the AE, dates that support a temporal relationship between the product and the event, and relevant laboratory results, were often either conflicting or missing [14]. Additionally, they found that in more than half of such reports, the date of the laboratory test results preceded the date of AE occurrence and the date of suspect drug use, occasionally by years. Another limitation is that the discussed methods focused on collecting data for the initial report and didn’t facilitate the collection of follow-up information. It is uncommon for AE reports to be of sufficient quality to make a proper assessment, as demonstrated by Durrieu et al. who found that only 12.7% of reports were well documented [15], so having the ability to also get timely, highly focused bespoke and relevant follow-up information is critical. Moreover, what data is important for safety assessment varies enormously by drug, AE, and drug-AE pair. Enabling PVEs who know exactly what important data is missing from the initial report to specifically ask the healthcare provider (HCP) for critical missing data, which may not even be available at the time of the initial AE report (e.g., bilirubin levels post drug discontinuation in a suspected drug induced liver injury report to confirm a positive dechallenge etc.), would not only help to create efficiencies for both parties but will also give the highest possible clarity to the AE report. In the assessment of causality Edwards et al. [16] established that three index cases, or their equivalent, are necessary to determine causality. These index cases can be substituted with a mix of “substantial” or “feasible” cases, but it's widely recognized that one needs to have at least one index case. In this context, the total number of cases needed would often be < 10.

Previous studies also focused on the collection of safety information from the healthcare professional but did not offer any information in return. The importance of this quid pro quo was highlighted by the Aster study that found 18 (78%) of participants said they would like to view aggregate national data from the FDA about similar events [13]. Although studies to date highlight the potential value of digitizing the AE process, as Dal Pan pointed out “the increasing availability of informatic systems holds much promise for pharmacovigilance—though, in some cases, a lack of relevant clinical or product details limits the utility of data systems [17].” To our knowledge, there have been no studies that evaluate the possibility of using digital technologies, such as EHRs, for near real-time bi-directional communications (both initial and follow up) between PVEs and HCPs for the exchange of AE information to support causal assessment.

The need is to ensure that PVEs get the right information that may not be obvious to the HCP, who is not trained in PV. At the same time, facilitating HCP participation by leveraging commonly used digital technologies to minimize the reporting burden and avoiding requesting information that, while potentially increasing the amount of data available for review, will have insignificant impact on PV understanding. The objective of our study was to demonstrate that by leveraging digital technologies iteratively with PVEs central to the process throughout, that it was possible to improve the timeliness and quality of safety data collection leading to “high-definition” actionable safety insights that are unlikely to have been attained otherwise.

## Materials and Methods

### EHR Platform

This proof of concept (PoC) was implemented on the Practice Fusion (PF) EHR platform and EHR database (Practice Fusion, San Francisco, CA, USA) among consenting practices as the Adverse Event Deep Dive (Add) program. The platform is currently in use at over 30,000 sites, mostly single-provider or small group practices, in all US states, representing approximately 6% of ambulatory care among primary care and specialist practices in the USA (https://www.practicefusion.com/about/). The PF EHR patient population is described as comparable to the overall US population in terms of age, gender, and geographic location [18].

### Selection of In-Scope Drug-Event Pairs/AEIs

Medications for the PoC were selected based on their prevalence of prescribing within an ambulatory care setting as reported in the Centers for Disease Control ambulatory and hospital care statistics, in combination with the product class having a well-documented adverse reaction which could be systematically screened [19].

#### Use Case # 1 (Add-mAbs)

Utilizing these criteria, interleukin (IL) inhibitors, Anatomical Therapeutic Chemical (ATC) code L04AC, for the treatment of dermatologic autoimmune disorders and serious infections were selected for the initial implementation (Supplemental Table 1). After study start, the list of IL was subsequently expanded to also include code L04AB to increase participation. Serious infections were the AEs of interest and defined as all infections that appear in the European Medicines Agency Important Medical Events list based on Medical Dictionary for Regulatory Activities preferred terms (MedDRA PTs) for medical conditions that are serious and usually drug-related[20]. These MedDRA PTs were mapped to an appropriate International Classification of Diseases – Tenth Edition (ICD-10) code.

#### Use Case # 2 (Add-Xabans)

After study start, Xabans (ATC code B01AF) and serious bleeding were added to increase participation. Serious bleeding events were defined based on ICD-10 codes included in Joos et al. 2019[21].

### HCP Recruitment, Consenting and Enrollment Process

HCPs on the platform with a record of prescribing an in-scope product were presented with banner notifications on the Add program. Clicking on the notification directed the HCPs to the program enrollment page that described the program and presented a consent form for enrolling the practice. HCPs could also access the program enrollment page via a tile on their practice dashboard. The vendor’s Clinical Affairs team (VCA), acting as a data intermediary, maintained a list of practices consenting to participate in the PoC to facilitate downstream follow-up and data exchange. The PoC program was monitored on an ongoing basis to evaluate HCP recruitment and AEI activity. A number of strategies/activities were implemented to increase participation (Supplementary Text).

### Operational Process

#### Identify Adverse Events of Interest (AEI)

An AEI was defined as when an HCP at a consented practice entered an ICD-10 matching one of the two use cases and the same diagnosis code (Supplemental Table 2) was not present in the 30 days prior and the date of entry was within the drug-specific period of risk (e.g., 90 days). An automated script was run by the vendor every 24 h to identify AEIs among consented practices. Once the AEI was identified the VCA would engage the HCP via secure messaging in the EHR. Information accessed by the VCA had the minimum necessary personally identifiable information (PII) to support interaction with the HCP, however this PII was not shared with anyone outside of the EHR provider, which was consistent with their normal operating practice.

#### Initial Communication

The initial communication was sent within 2 h of identifying the AEI and asked whether the HCP believed that there was a reasonable chance the event could be related to the use of the in-scope drug. If their response was yes, the HCP was requested to provide additional information on the AEI including (1) to confirm the diagnosis and differentiate between a symptoms-based diagnosis (provisional) vs a definitive diagnosis (2) ensure that there is reasonable temporality between administration of medication and occurrence of the AEI, and (3) establish any initial intrinsic and extrinsic risk factors that may be associated with the event. If their response was no, then the interaction was stopped and the HCP was instructed to ignore the subsequent questions. In addition, FDA Adverse Event Reporting System (FAERS) public dashboard information related to the AEI was provided to the HCP as a part of the initial communication. The VCA acted as a trusted intermediary to facilitate review by the PVE and reduce burden on the HCP by removing any PII, and extracting relevant information from the patient’s medical record based on the criteria defined a prioi by the PVE deemed to be relevant to the assessment of the AE, this information was sent to the PVE via secure messaging. VCA’s access to the patient’s medical records forms part of existing operating procedures established by the EHR vendor.

#### Follow-up Communication

The PVEs assessed the information received from the VCA within 2 h of receipt and, if necessary, requested additional information from the HCP, via the VCA, to obtain case-specific information required to properly assess causality. Once the VCA received the follow-up request it was reviewed to ensure appropriateness of the request and subsequently forwarded the request to the HCP within 2 h of receipt. Similar to the initial communication once the VCA received the follow-up response from the HCP, it was reviewed by the VCA and then forwarded to the PVEs within 2 h. If needed, the program allowed for a second round of follow-up.

#### End of Program Survey

After the close of the HCP follow-up communication, the VCA surveyed the HCP within 72 h for feedback on perceived value of the bi-directional communication approach and if the HCP found it helpful to receive the aggregated FAERS data on the drug of interest.

### Study Measures

#### Operational Measures

Key program outcomes included the number of AEIs identified and number of completed HCP interactions. In addition, elapsed time for each step in the communication process was collected.

#### PVE AEI Assessment

Three PVEs evaluated the totality of the responses from the HCP, initial and bespoke follow-up, and determined if sufficient data was present to assess causality. Concordance across all 3 PVEs was required to determine the data enabled a proper causality assessment.

### Governance, Oversight, and Ethical Compliance

This program leveraged existing HCP communication channels within the platform. The VCA acted as a Trusted 3rd Party to facilitate bidirectional data exchange with the pseudonymization of personal and medical information; this ensured the patients’ and HCPs’ anonymity was maintained and that their identities were protected and not shared with the PVEs or those that do not have routine access to the platform. The VCA had access to the minimum necessary HCP contact information and PII pertaining to the AEI to support interaction with the HCP; this was limited to use only by the VCA directly supporting the program. In addition VCA existing access to the patients’ medical record via the EHR vendors existing operating practice was leveraged to facilitate the collection of relevant safety information to support assessment and reduce burden on the HCP.

The VCA also ensured the appropriateness of the communication by ensuring that responses from the HCP or requests from the PVEs did not impact clinical decision support, contain requests for transfer of value, contain PII information, or relate to communications that would be considered outside the scope of PV. A robust mechanism was integrated into the program to ensure that any communications deemed inappropriate was not delivered, documented and communicated by the VCA to the relevant party.

In addition, whilst there are no specific regulatory obligations to report safety information for drugs for which the pharmaceutical company is not the Marketing Authorization Holder, appropriate due diligence was undertaken to ensure AEs were reported to the relevant drug manufacturer within 24 h of notification by the VCA. Suspected AEs may have come from the in-scope AEI or from additional information received directly from the HCP through the AE communication process.

This study underwent Institutional Review Board (IRB) review by Advarra, Inc. (protocol#: Pro00066412) prior to commencing, to ensure ethical and privacy compliance. This research was determined to be exempt from IRB oversight.

## Results

### Enrollment

**Fig. 1:**
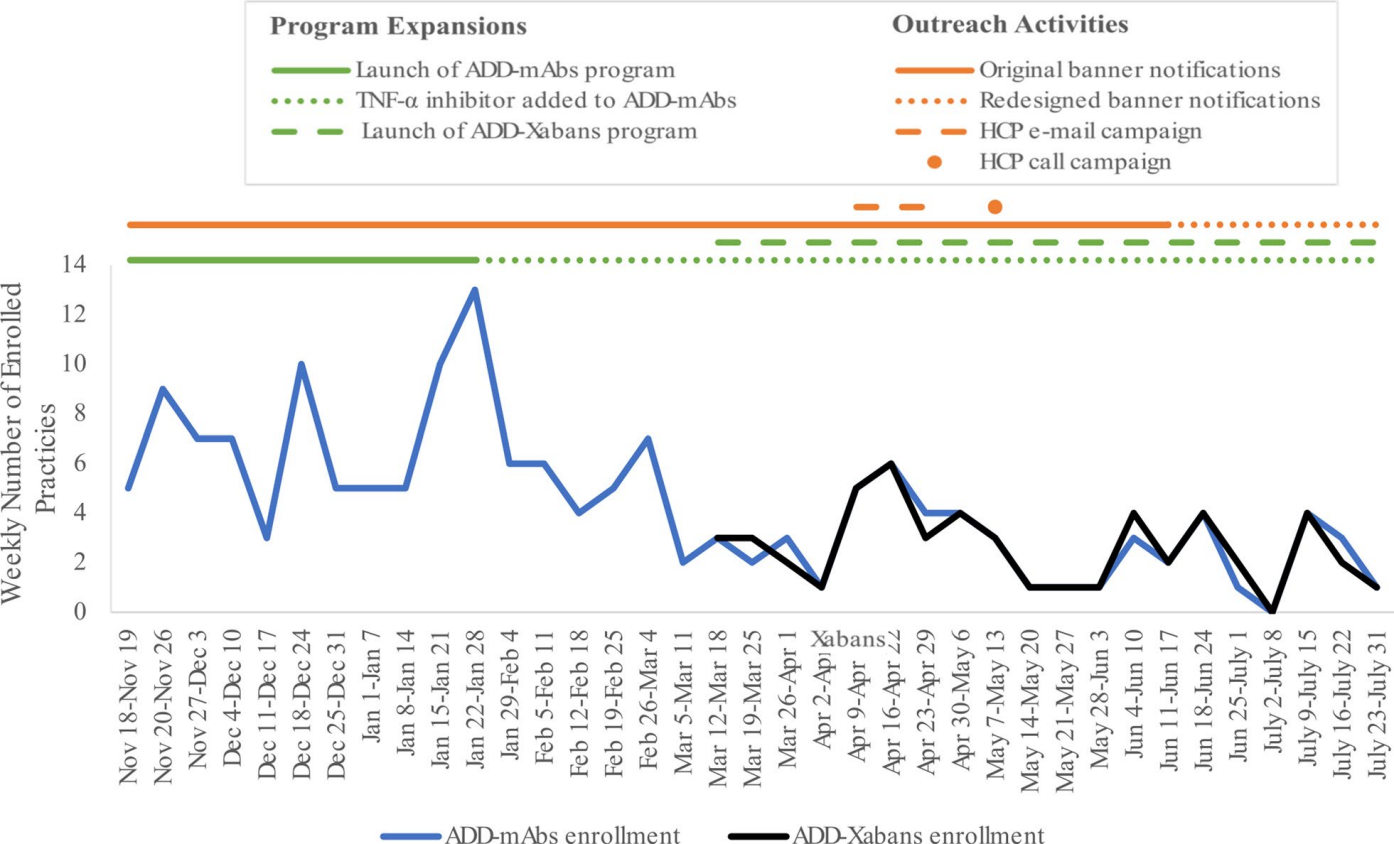
Practice enrollment and enrollment-related activities over the course of the program. Add-mAbs: Adverse Event Deep Dive program with interleukin inhibitors as drug class of interest; Add-Xabans: Adverse Event Deep Dive program with direct factor Xa inhibitors as drug class of interest; *HCP* healthcare provider

Ongoing recruitment for participation in the Add program occurred over the course of the program from November 2022 through July 2023 with additional expansion and outreach activities being implemented over the course of the program to increase HCP participation (Fig. 1). Results related to enrollment activities aimed at increasing HCP participation are presented in Supplementary Text.

In total, 43,140 HCPs were presented with banner ads for program enrolment with just over a thousand (n = 1009) HCPs enrolling, the majority enrolled in both the mAbs and the Xabans programs. In addition, HCPs also had the option to learn about the program and enroll through their practice dashboard without being specifically targeted for inclusion via the banner campaigns. On average, each targeted practice had 6 HCPs. The Add-mAbs program saw an average weekly enrollment of 26 HCPs, while the Add-Xabans program enrolled an average of 16 HCPs per week. Mean (standard deviation) weekly enrollment was 4.3 (2.9) practices per week for the Add-mAbs program and 2.6 (1.6) practices per week for the Add-Xabans program.

### AEIs

**Fig. 2:**
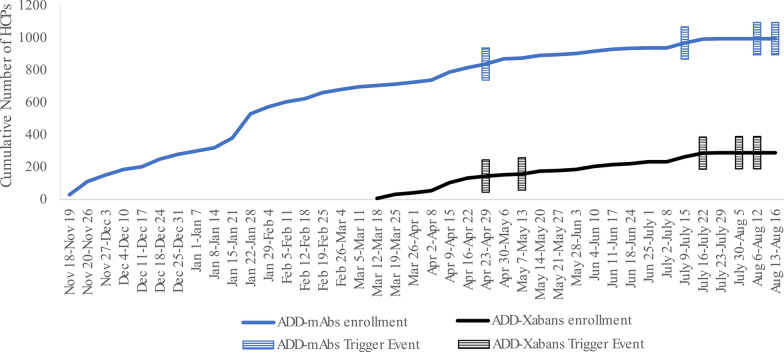
AEI occurrence by program. *HCP* healthcare provider, *AEI* adverse event of interest, *Add-mAbs* adverse event deep dive program with interleukin inhibitors as drug class of interest; Add-Xabans: Adverse Event Deep Dive program with direct factor Xa inhibitors as drug class of interest

The program was launched on November 18, 2022, with the first of nine AEIs occurring in April 2023 (Fig. 2). Four events were recorded among patients prescribed mAbs and five events were recorded among patients prescribed Xabans. Contact could be initiated with HCPs for seven of the nine events, of which three of the contacted HCPs did not respond leaving four events available for initial assessment. Detail on initial interactions for each AEI including program, date, and communication metrics captured by the VCA are provided (Table 1). HCPs for all four AEIs available for initial assessment responded to the questions from the VCA to assess AEI status. Of these four events, two were indicated as suspected AEs by the responding HCP (see details below). Three responses from HCPs were timely, in less than 5 h, while one took approximately 3 days (Table 1).

**Table 1:** Summary of initial interactions for AEIs

Event	Program	Date	Time trigger to HCP contact (hours)	HCP indicated eventas suspected ADR	Time HCP to answer questions (hours)	VCA event status
1	Add-mAbs	4/24/2023	0.1	No	3.0	Not in scope
2	Add-Xabans	4/25/2023	0.2	Yes	72.3	In scope
3	Add-Xabans	5/9/2023	< 0.1	No	4.3	Not in scope
4	Add-mAbs	7/12/2023	0.1	No response	N/A	N/A
5	Add-Xabans	7/21/2023	< 0.1	No response*	N/A	N/A
6	Add-Xabans	8/1/2023	0.1	Yes	2.6	In scope
7	Add-mAbs	8/9/2023	Unable to connect	N/A	N/A	N/A
8	Add-Xabans	8/11/2023	< 0.1	No response	N/A	N/A
9	Add-mAbs	8/13/2023	Unable to connect	N/A	N/A	N/A

*HCP responded that they had not had a recent interaction with patient

*Add-mAbs* Adverse Event Deep Dive program with interleukin inhibitors as drug class of interest, *Add-Xabans* Adverse Event Deep Dive program with direct factor Xa inhibitors as drug class of interest, *ADR* Adverse drug reaction, *VCA* Veradigm Clinical Affairs, *HCP* Healthcare provider

**Fig. 3:**
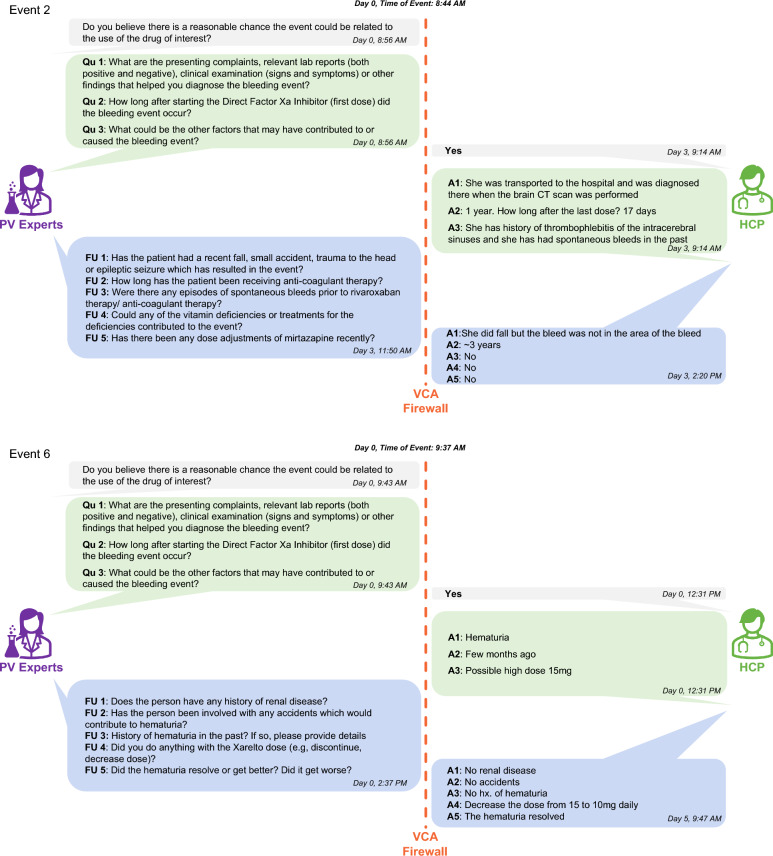
Clinical assessment communications and detail for two suspected AEIs. *FU* follow-up, *A* answer, *VCA* veradigm clinical affairs, *PV* pharmacovigilance, *HCP* healthcare provider

Additional detail on the timing of the communication process for follow-up of the two suspected AEs is provided (Fig. 3). The PVEs follow-up assessment responses to confirm each suspected AE were conducted in approximately two hours. HCPs for both suspected AEs responded to program communications related to establishing AE status with timing from two hours to several days. On average, it took 20.6 h to receive initial AEI information and 58.8 h to receive follow-up information. The two suspected AEs were confirmed by both the VCA and the PVE, and AEs were reported to each manufacturer accordingly. One of these two HCPs responded to the final program survey and that took about a week to complete (Fig. 3).

### Completed Interaction

Initial questions with HCP responses and customized follow-up questions are provided for each of the two completed HCP interactions (Fig. 3).

### Completed Interaction #1

This interaction involved a bleed secondary to Xaban use. In the initial interaction the HCP was able to confirm the event, subdural haemorrhage, was a potential adverse event, the symptoms and diagnostic tests used to confirm the event (CAT scan), the time to onset (1 year), as well as other factors that could have contributed to the event (history of spontaneous bleeds in the past). After review by the PVEs, 5 additional follow-up questions were sent to the HCP via the VCA:

1. Did the patient recently have a fall, accident, or trauma to determine if there was an alternative explanation to the bleeding.
2. Verify the patient’s anticoagulation history. If the patient had been on other anticoagulants, then the HCP could be asked about bleeding events with other drugs (see Question 3 below).
3. Did the patient have any bleeding history with use of other anticoagulants as a way to assess if the patient is prone to bleeding with anticoagulants.
4. The HCP was asked about vitamin deficiencies as some vitamins (e.g., vitamin K) play a role in the clotting cascade.
5. Had there been a dose adjustment with the patient’s mirtazapine as there is a potential drug-drug interaction with some antidepressants and Xabans.

In the responses to the bespoke questions above (see Fig. 3) the HCP did confirm the patient recently fell but stated the location of the injury was not in the area of the bleed which allowed the PVEs to dismiss the fall as an alternative explanation for the bleed. Additionally, the HCP confirmed there were no vitamin abnormalities which could have contributed to the patient’s history of spontaneous bleeding with anticoagulants [22]. Both of these questions are highly contextualized within the specific drug event pair and unlikely to be reported and/or collected upon follow-up using more traditional pharmacovigilance techniques.

### Completed Interaction #2

This interaction involved a bleed secondary to Xaban use. In the initial interaction the HCP was able to confirm the event, hematuria, was a potential adverse event, the presentation of the event was blood in the urine, and the time to onset (a few months ago), as well confirm the patient was on a high dose of anticoagulation (15 mg). After review by the PVEs, 5 additional follow-up questions were sent to the HCP via the VCA:

1. Did the patient have a history of renal disease, as the event was blood in the urine, to rule out background disease as a potential alternative explanation.
2. Had the patient had any accidents that could have offered an alternative explanation of the event.
3. Did the patient have a history of the event in the past to assess if the patient was predisposed to the event.
4. Did the HCP reduce the dose of anticoagulation since the patient was on a high dose.
5. Did the event resolve after reducing the dose to confirm a positive dechallenge.

Time to onset is uniquely important for anticoagulants as major bleeds with some anticoagulants are higher within the first year of initiation [23]. Additionally, the history of renal disease is also important, in addition to being a potential alternative explanation of the bleed, given many anticoagulants rely on the kidney for elimination to varying degrees [24]. As a result, a decrease in renal function could have caused an accumulation of the anticoagulant therefore increasing the risk of the bleed. Both of these points are specific considerations for pharmacovigilance for this drug-event pair and unlikely to appear important to someone not specifically trained in the intricacies of drug safety for this specific drug-event pair. As a result, it’s very unlikely these important elements will be initially reported or collected upon follow-up using more traditional pharmacovigilance techniques.

### PV Expert Assessment

For both of the AEIs all three PVEs reviewed the totality of the data provided, from the initial set of questions and the bespoke follow-up, and determined that sufficient data existed to make a proper assessment of causality.

### HCP Survey

One of the two HCPs responded to a brief seven question post program survey. The HCP responded to all survey questions. While the HCP did not find having the ability of near real-time bidirectional exchange of safety related information with the PVE potentially valuable, they did indicate that they would participate in similar activities in the future. The HCP indicated that they were not previously aware of the FAERS information and found the FAERS information included in the initial interaction to be helpful. The HCP also responded that outside of this PoC they would not have reported this suspected AE to the drug manufacturer or the FDA.

## Discussion

Our results show that, as postulated, we were able to leverage digital tools, such as the EHR, to improve the timeliness, quality, and uniqueness of safety data collection leading to “high definition” actionable safety insights that are unlikely to have been attained otherwise.

There were several strengths to our methodology. First, we were able to show significant improvement in the timeliness of the exchange of AE information. Using traditional methods, the average time from AE occurrence to receipt of follow up was 106 days. In our PoC, we were able to demonstrate the 4 completed interactions (2 completed interactions including follow-up and 2 completed interactions that indicated the event was not an AE) took about 2 days on average, an overall time savings of 97%. Even when looking at just the 2 completed interactions the time savings was still 96% (10–12).

Second, we were able to demonstrate qualitatively that the resulting data, from the initial questions and bespoke follow-up, was significantly better than data obtained through the current process. In our study, both interactions (for which we received confirmation of the AE and answers to the initial and bespoke follow-up questions) were of sufficient quality for proper assessment of the event. For comparison, the study by Durrieu et al. found that only 12.7% of reports were well-documented (15). Additionally, our study demonstrated the value of PVEs having the ability to engage in near real-time, “active discussion” with HCPs. For example, the initial questions were focused, drug-event pair specific which allowed the HCP to provide the exact information the PVEs needed to perform an initial assessment as well as formulate highly focused follow-up questions to collect any missing information. With respect to the follow-up, in the two use cases both HCPs were asked about potential accidents or injuries that could have caused the bleeding, in one instance the HCP confirmed no accident whereas the other HCP confirmed the patient did have a recent injury, however the location was not in the area of the bleed. Additionally, the contextualized discussion led to unique insights that are contextualized to the specific drug event pair. For example, collecting history of renal disease to not only rule out an alternative etiology of the event but also assess proper drug elimination from the body. This highly focused exchange of information isn’t possible with the current process where PVEs are limited to whatever information the reporter wants to provide with the initial report, without influence from safety experts, and is further limited by the time it takes to collect follow-up, which can introduce recency bias into the additional data provided, if any.

Finally, our methodology addresses barriers to HCP participation. First, HCP burden was minimized by leveraging existing data from structured fields in the EHR and asking only the highly focused relevant questions that needed to be asked from a PVE perspective, specific to the exposure-outcome of interest. Secondly, our method raised awareness by providing summary data, such as FAERS, back to the HCP. Thirdly, it facilitated communication with the drug manufacturer, providing the HCP with the opportunity to engage with the PVEs.

There were limitations to our study. Unfortunately, with only 9 trigger events identified in 9 months and with only 4 completed interactions (2 completed interactions including follow-up and 2 completed interactions that indicated the event was not an AE), the number of completed interactions was lower than expected. It should be noted however that no trigger events were identified in the first 5 months, with all nine trigger events occurring in the last 4 months of the study with 6 in the last 6 weeks. This suggests a potential lead time may be required to recruit enough HCPs to reach a critical mass to enable this capability. If the study were expanded to include other EHR providers, we would need to enroll sufficient EHR providers to cover 30% US market share (5—fold increase) to achieve sufficient HCP participation for 10 completed interactions including follow-up, comparable to the magnitude of cases as detailed by Edwards et al. This level of participation, given the higher quality of data, would be sufficient to justify most regulatory actions. However, this extrapolation would be influenced by many factors e.g. frequency of prescribing of the drug and incidence rate of the AE of interest.

Second, our study was based on a US-based EHR, where the market is highly fragmented, and we only included 2 well known use cases. We are not sure if this capability would be generalizable outside of the US, although we don’t see any reason why not. Also, the EHR provider we used is focused on smaller independent practices many of which are primary care, so if this capability would work as well with larger practices and/or non-primary care providers is unknown, however there are no obvious reasons why this capability wouldn’t generalize to other EHR providers or similar digital communication tools based on the healthcare delivery setting. Finally, using such well known AEs as use cases may have prevented HCPs from consenting to participate. Given the current time pressures on healthcare it is not unreasonable to assume HCPs would allocate their time for value-add activities, such as less well known adverse events, that would most benefit the patient.

An area for potential improved participation might be to compensate HCPs for their participation. Although protecting patients is of paramount importance it is important to also realize the business side of medicine and the strained resourcing model that currently exists. If payments could be made in a transparent, ethical way then this may help facilitate participation, albeit in a potentially non-random way.

Although our study demonstrated value of the overall concept, there are a number of open questions that remain. The first question is around putting into place guardrails for a scaled implementation to ensure the information shared was only within the context of drug safety. These guardrails would define appropriate use to complement existing PV activities to support qualitative signal assessment for key safety concerns. These key concerns would likely represent specific drug-event pairs, such as those discussed in the products risk management plan and/or those that can significantly impact a drug’s benefit risk. Once this capability has yielded enough quality reports so a safety assessment can be reliably made, there maybe limited value in collecting any further data in this manner to inform benefit-risk. As it only takes a minimum number of high quality reports to support safety assessment we anticipate the volume of specific reports to be minimal, lower than 1.67 reports/HCP/month, based on the ASTER proof of concept study which enrolled 26 clinicians and reported 217 AEs over a 5 month period. Note that ASTER study was not restricted by Drug-Event pair, nor was it limited to key safety concerns therefore our estimate is likely to be conservative in comparison to restriction by drug-event pair. As an example of the rarity of a serious outcome, the incidence of myocarditis associated with covid-19 vaccines in the US population was estimated to be 1.27 per 100,000 patient days of exposure in the strata most impacted (men aged 18–25 years), and the longest risk period was 42 days, limiting the period of monitoring [25].

Although this capability offers significant potential to improve drug safety, it comes at the risk of it being leveraged inappropriately**.** Ensuring that there are no commercially related discussions as well as avoiding involvement in the actual delivery of healthcare is critical. We have shown how this can be done and that it was effective in this study, but moving forward there is a need to establish best practices within the healthcare, pharmaceutical, and regulatory communities to establish clear operational considerations to ensure this valuable communication channel stays open for the benefit of patients.

The next question is around the capability, in general. Although we have described our experiences using it for drug safety, this capability could generalize to any prospective data collection where timely interactive discussion is required or more likely to elicit key information while minimizing HCP/and patient burden. For example, could others leverage this capability to allow for increased clarity of benefits to ensure the most up to date benefit-risk profile is available. One risk of expanding beyond drug safety is that we can quickly overload already busy HCPs. Although our study demonstrated the minimal time required for HCP participation, an increase in the number of requests could quickly become burdensome to HCPs and discourage participation.

## Conclusion

We demonstrated the feasibility of implementing a digital, compliant, bidirectional clinical communication channel from the point of care between HCPs and PVEs that can complement existing PV activities by providing near real-time, HCP-validated AE data that was superior to data that would be normally received through traditional channels. As with any emerging technology there are still many open questions that need to be addressed to maximize the potential this capability may offer the PV community. Additionally, there is a clear need to agree and harmonize on best practices to ensure appropriate utilization, so this capability could be scaled while ensuring patients are protected.

## Supplementary Information

Below is the link to the electronic supplementary material.

Supplementary file1 (DOCX 254 KB)

Supplementary file2 (PDF 12 KB)

Supplementary file3 (XLSX 65 KB)

## Data Availability

The datasets generated and/or analyzed during the current study are not publicly available but are available from the corresponding author on reasonable request.
