# Supplementary material for: Testing the Feasibility of a Digital Point of Care Solution for the Trusted Near Real-Time Bidirectional Exchange of Novel and Informative Adverse Event Information
Source: Ther Innov Regul Sci. 2024 Nov 17;59(1):124–34. doi: 10.1007/s43441-024-00711-9 (PMC11706924; doi:10.1007/s43441-024-00711-9)
Supplement: Supplementary file 1 — Supplementary file1 (DOCX 254 KB) [file 43441_2024_711_MOESM1_ESM.docx]

**Title:** Testing the Feasibility of an Electronic Health Records Based System for the Trusted Near Real-Time Bidirectional Exchange of Adverse Event Information

**Journal Name:** Therapeutic Innovation & Regulatory Science

**Authors:** Greg Powell^1^, Vijay Kara^2^, Daniel Naranjo^1^, Mangesh Kulkarni^1^, Kerri Best-Sule^1^, Trinka Coster^3^, Machaon Bonafede^4^, Shruti Gangadhar^4^, Lee Kallenbach^4^, Andrew Bate^2^

**Affiliation:** 1. GSK, Durham NC, USA; 2. GSK, London, UK; 3. Pharmacocybernetics, LLC., Potomac MD, USA; 4. Veradigm Inc., Chicago, IL, USA

**Corresponding Author:** Greg Powell ([gregory.e.powell@gsk.com](mailto:gregory.e.powell@gsk.com))

***Methods***

***Program expansion and activities***

The PoC program was monitored on an ongoing basis to evaluate HCP recruitment and AEI activity. A number of strategies/activities were identified a priori and were available to implement to increase the number of available practices and level of participation if the expected enrollment rate or number of completed HCP interactions was below expectations. These included program expansion and activities to encourage program participation.

To expand the program, TNF-α inhibitors (L04AB), which have similar immunosuppressive effects and risk of serious infections as already included mAbs were added. The addition of TNF-α inhibitors almost doubled (increase by 90%) the number of HCPs eligible for participation (Implemented: January 24, 2023). The event of interest, serious infections, stayed the same. In addition, targeting a different HCP specialty, direct factor Xa inhibitors (B01AF) were also added. The addition of direct factor Xa inhibitors more than doubled (increase by >200%) the number of HCPs eligible for participation (Implemented: March 12, 2023). The event of interest was serious bleeding.

To encourage program participation an HCP email campaign to raise visibility of the program and support practices with the enrollment process was conducted (Implemented: April 12-24, 2023). Similarly, an HCP call campaign to practices was conducted to obtain information on roadblocks/reasons for non-participation (Implemented: May 11-12, 2023). In addition, the banner notifications were presented in a simpler redesigned format to eligible HCP practices (Implemented: June 16, 2023).

***Results***

***HCP Enrollment***

HCP awareness and recruitment was conducted through banner notifications in the EHR that contained an embedded link that, when clicked, directed HCPs to the program consent and enrollment page. Overall, 19,889 clicks were registered over the course of the program resulting in a 0.3% average click-through rate (CTR) for the program (Figure S1). A redesign of the banner notification in late June led to an increased CTR (Figure S1) but did not have a direct impact on enrollment (Figure 1). In addition, outreach through email was conducted in mid-April to foster program participation. Altogether, of the 75,778 emails that were sent out the click to open rate was 2.6%. A slight increase in enrollment was observed for both programs with the roll out of the email campaign. For a subset of practices that did not enroll via the email outreach, qualitative telephone interviews with follow-up emails were conducted with practice managers to gauge program interest. Six of the practices responded with information about non-participation, (interested, not a good fit, not interested, did not recall clicking on the program enrollment link).


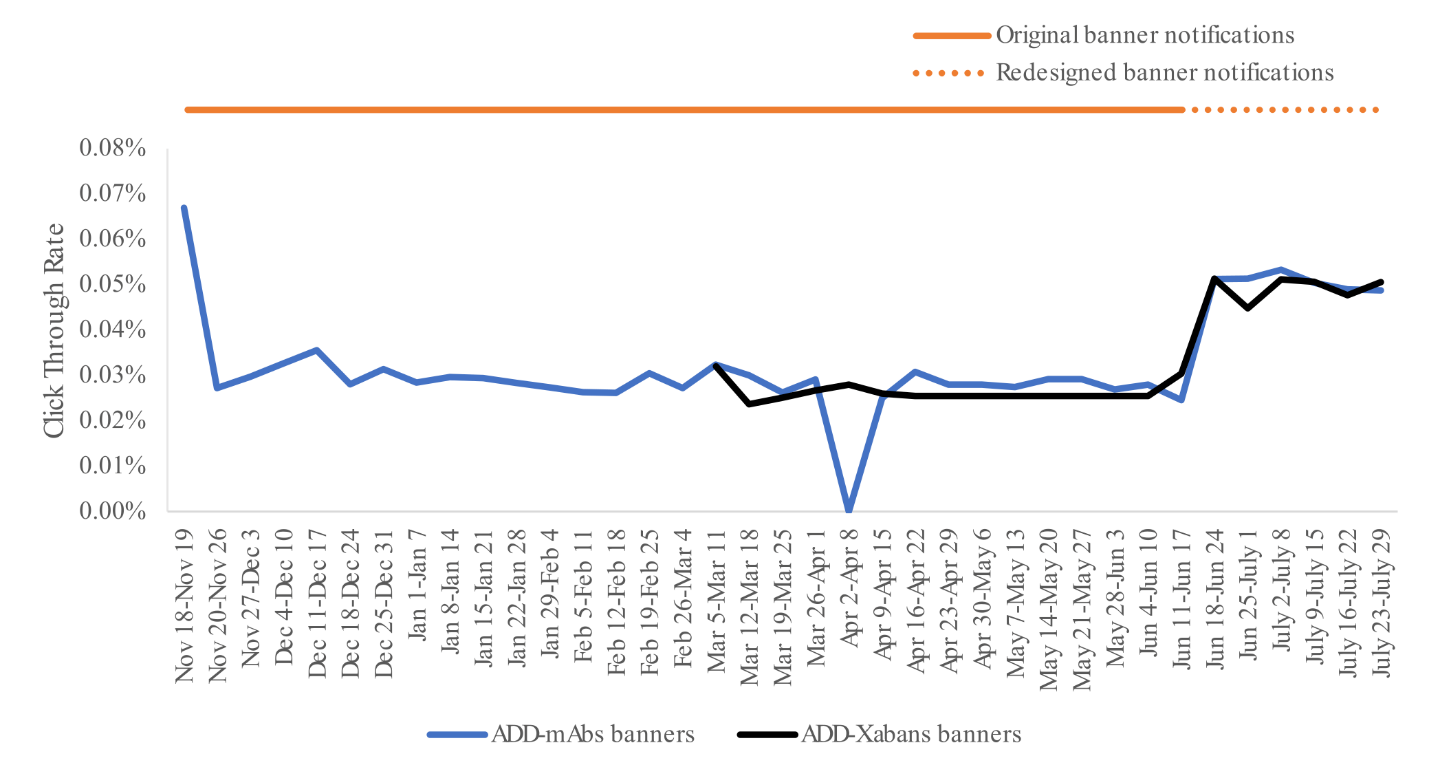


**Fig S1.** Click-through rate (CTR) for the Add-mAbs and Add-Xabans Banner Notification Campaigns. CTR: The ratio of clicks on the banner notifications to the number of times the notifications were presented; Add-mAbs: Adverse Event Deep Dive program with interleukin inhibitors as drug class of interest; Add-Xabans: Adverse Event Deep Dive program with direct factor Xa inhibitors as drug class of interest.
