# Supplementary material for: Testing the Feasibility of a Digital Point of Care Solution for the Trusted Near Real-Time Bidirectional Exchange of Novel and Informative Adverse Event Information
Source: Ther Innov Regul Sci. 2024 Nov 17;59(1):124–34. doi: 10.1007/s43441-024-00711-9 (PMC11706924; doi:10.1007/s43441-024-00711-9)
Supplement: Supplementary file 2 — Supplementary file2 (PDF 12 KB) [file 43441_2024_711_MOESM2_ESM.pdf]

Title: Testing the Feasibility of an Electronic Health Records Based System for the Trusted Near Real-Time Bidirectional Exchange of Adverse Event Information

Journal Name: Therapeutic Innovation & Regulatory Science

Authors: Greg Powell[1], Vijay Kara[2], Daniel Naranjo[1], Mangesh Kulkarni[1], Kerri Best-Sule[1], Trinka Coster[3], Machaon Bonafede[4], Shruti Gangadhar[4], Lee Kallenbach[4], Andrew Bate[2]

Affiliation: 1. GSK, Durham NC, USA; 2. GSK, London, UK; 3. Pharmacocybernetics, LLC., Potomac MD, USA; 4. Veradigm Inc., Chicago, IL, USA

Corresponding Author: Greg Powell (gregory.e.powell@gsk.com)

Supplementary Table 1. In-scope drugs

**Program**	**Drug Class**	**Drug name**
Add_mAbs	Interleukin inhibitors	Tocilizumab (Actemra®), Sarilumab (Kevzara®), Guselkumab (Tremfya®), Tildrakizumab (Ilumya®), Risankizumab (Skyrizi®), Secukinumab (Cosentyx®), Ustekinumab (Stelara®) and Ixekizumab (Taltz®)
	Tumor necrosis factor alpha inhibitors	Infliximab (Remicade®), Adalimumab (Humira®), Certolizumab pegol (Cimzia®), and Golimumab (Simponi®)
Add_Xabans	direct factor Xa inhibitors	rivaroxaban (Xarelto®), apixaban (Eliquis®) and edoxaban (Savaysa®)
